# A North Atlantic synthetic tropical cyclone track, intensity, and rainfall dataset

**DOI:** 10.1038/s41597-024-02952-7

**Published:** 2024-01-25

**Authors:** Wenwei Xu, Karthik Balaguru, David R. Judi, Julian Rice, L. Ruby Leung, Serena Lipari

**Affiliations:** Pacific Northwest National Laboratory, Richland, 99354 USA

**Keywords:** Natural hazards, Atmospheric dynamics, Energy security

## Abstract

Tropical Cyclones (TCs) cause significant socio-economic damages to the US and Caribbean coastal regions annually, making it important to understand TC risk at the local-to-regional scales. However, the short length of the observed record and the substantial computational expense associated with high-resolution climate models make it difficult to assess TC risk using either approach. To overcome these challenges, we developed a database of synthetic TCs using the Risk Analysis Framework for Tropical Cyclones (RAFT). The database includes 40,000 synthetic TC tracks, along-track intensities and storm-induced precipitation. TC tracks generated in RAFT are in reasonable agreement with the observed spatial distribution of TC tracks and basin-scale TC statistics. Specifically along the coast, spatial variations in TC crossing probability and extreme winds upon landfall are well-reproduced by RAFT with R-squared values of 0.81 and 0.73, respectively. In summary, the synthetic TC database constructed with RAFT provides a reasonable pathway for the robust assessment of North Atlantic TC wind and rainfall risks.

## Background & Summary

Tropical cyclones (TCs) are among the most destructive natural hazards for the North and Central American regions, and are responsible for 71% of fatalities and 78% of economic losses due to weather extremes over the period 1970–2019^1^. Within the United States (US), they have caused over 6,500 fatalities and inflicted economic damages of about $1.1 trillion over the last 40 years^2^. This makes it critical to understand the risk associated with Atlantic TCs at the regional-to-local scales, where damages manifest most profoundly^3^. Using observations to quantify TC risk is difficult, considering the short length of the reliable TC record and the fact that annually only 1–2 storms make US landfall on average^4^. While high-resolution climate models that can explicitly resolve TCs may provide a viable alternative, simulating a large number of storms using such models typically incurs a high computational cost.

To address these issues of data-scarcity and computational expense, a suggested approach has been to generate a synthetic TC record to supplement the historical record, providing a more complete picture of TC characteristics at regional-to-local scales. This approach was likely first introduced by Vickery *et al*.^5^, who generated a set of synthetic storm tracks in the Atlantic basin using a fully statistical approach. This method was improved upon in 2006 by Emanuel *et al*.^6^ by coupling two track generation models with a deterministic intensity model, ensuring that storm intensity conforms to the underlying physics. A similar statistical-dynamical approach was adopted by Lee *et al*.^7^ to simulate synthetic global tracks with an environmental index-based genesis model and an auto-regressive intensity model. More recently, Bloemendaal *et al*.^8^ developed a purely statistical algorithm to generate synthetic TC tracks at the global scale by means of synthetic resampling. Another recent study^9^ coupled a statistical Markov renewal TC track model with a physics-based rainfall model to simulate TC rainfall over South Korea. Further, several of these studies have made their data publicly available^7,8^. However, the data provided only includes synthetic TC tracks and along-track intensities, and does not provide estimates of TC-induced rainfall over the North Atlantic region.

**Fig. 1:**
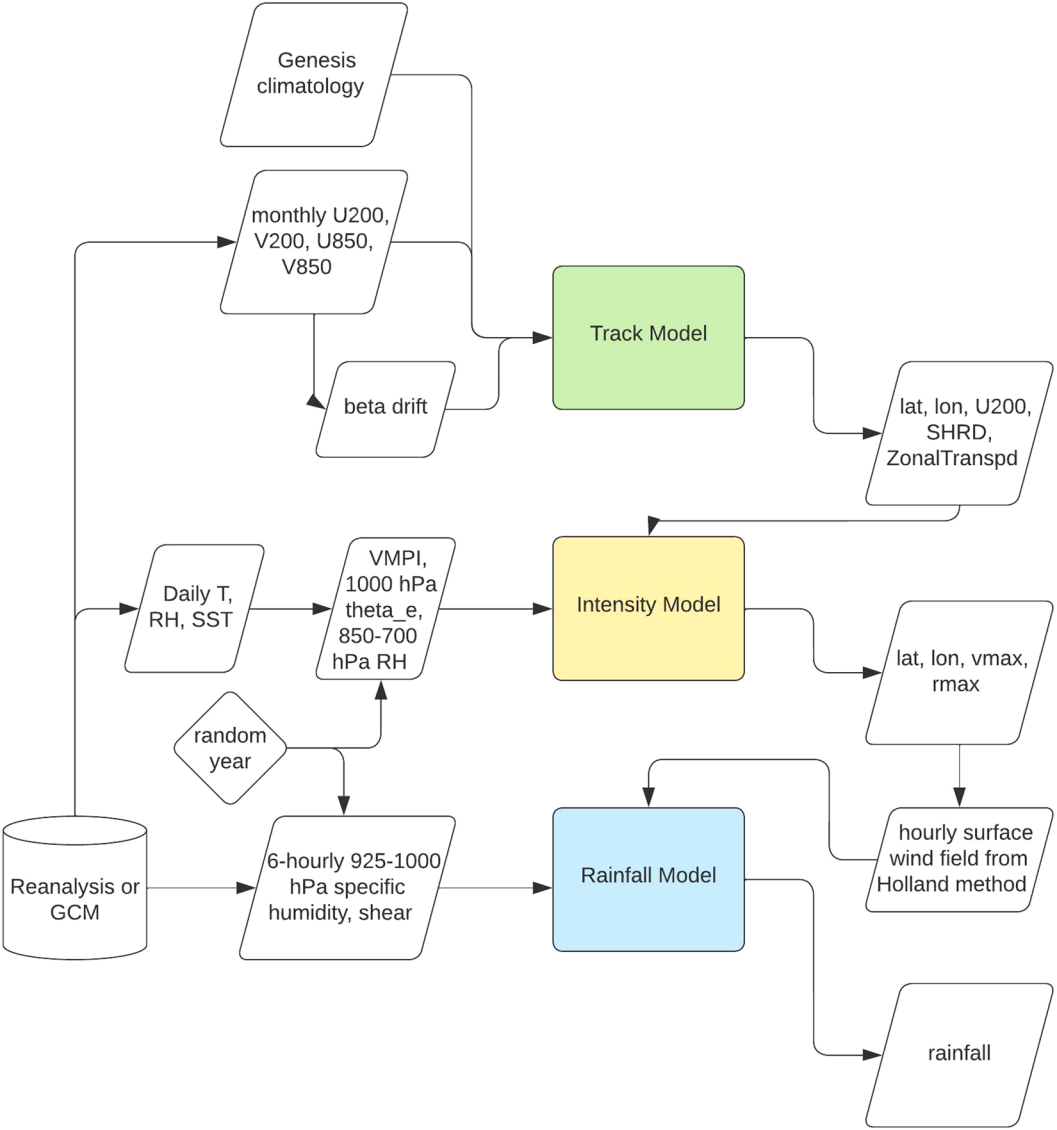
Overview of the dataflow for the synthetic TC dataset creation using RAFT.

While the high winds associated with TCs directly cause substantial damage to buildings and structures, storm-induced flooding and the generation of coastal storm surge additionally have devastating impacts on life and infrastructure. For instance, the torrential rain from TC Harvey (2017) resulted in catastrophic flooding in the Houston metropolitan area, estimated to have been responsible for economic damages of about $97 billion^1^. Later, in September 2018, TC Florence made landfall near Wilmington, North Carolina and became the ninth most intense hurricane to affect the US, largely due to extreme rainfall and flooding exacerbated by its slow progression^10^. This highlights the need for a TC database that includes precipitation in addition to winds to more holistically represent TC risk. To address this, we have developed a database of synthetic TCs using the Risk Analysis Framework for Tropical Cyclones (RAFT), a unified framework capable of simulating tens of thousands of TCs and their impacts based on the large-scale environment^11,12^. RAFT consists of several different components coupled into one cohesive system, as illustrated in Fig. 1. RAFT uses established methods for storm genesis^6^, track translation^6^, and precipitation^13,14^, while the intensity model is a novel deep neural network-based approach developed for this framework^12^. We believe the data generated by RAFT is unique and valuable to both the scientific community and the general public by nature of the unique combination of RAFT’s salient features, as outlined below:

- A variety of methods have been evaluated for each component of RAFT – many derived from established methods in the field of TC modeling – and we have selected those with the best balance between performance and computational efficiency. The resulting system utilizes a variety of techniques, from physical equations to linear models to deep neural networks.
- We include storm-induced rainfall in our dataset, which despite being one of the primary drivers of TC risk^2^ is generally not provided in other synthetic TC datasets. Since wind and rainfall parameters are generated together in a coupled approach, our method allows for a more consistent simulation and understanding of secondary risks such as compound flooding and power outages.
- RAFT is computationally efficient, allowing many thousands of storms to be generated in a reasonable amount of time. Specifically, it takes roughly 3 hours to generate 40,000 synthetic TCs using the track model and 5 hours to calculate intensity labels when run on a desktop with four Intel Xeon CPUs @ 2.80 GHz each and 32 GB of memory. This allows RAFT to be used for TC risk assessment at a fine resolution over large spatial and temporal scales.
- Importantly, RAFT can be run using simulated environmental conditions, such as those generated by climate models. It follows that RAFT can generate TCs representative of not just the historical or present climate, but potential future climates as well.

Using RAFT, we generated 40,000 synthetic TC tracks for the North Atlantic based on the current climate conditions obtained from ERA5^15^ reanalysis for the period 1979–2018. These climatic conditions – variables including large scale wind, sea-surface temperature, air temperature and humidity – are used to determine the various parameters of our synthetic storms. For each TC, we model its track, intensity, and rainfall pattern (0.08 resolution) at 6-hourly time steps. We make this data publicly available to facilitate a deeper understanding and further analysis of North Atlantic TC risk. While the RAFT framework has previously been applied to investigate the evolution of TC risk in the US^11^, here we provide a more thorough description of each of the framework components, perform an in-depth validation of the output, and discuss the constituent models’ limitations and potential for generalizability.

## Methods

### Track

After TCs are generated as a random draw from a Gaussian kernel-based probability distribution centered around historically observed TC genesis locations^6^, TC tracks are subsequently modeled using a beta-advection method following Marks *et al*.^16^ and Emanuel *et al*.^6^. Essentially, this method assumes that a TC track is governed primarily by the background steering flow, which is estimated as a weighted mean of the large scale wind at 850 hPa and 200 hPa levels. While Emanuel *et al*.^6^ uses 850 and 250 hPa winds, we chose to use 850 and 200 hPa winds as implemented in Marks *et al*.^16^ to be consistent with how wind shear is calculated within the intensity model. For generation of the synthetic zonal and meridional wind values at these levels, three components are used: the mean monthly winds in the zonal and meridional directions at 850 hPa and 200 hPa, the covariance component between these four wind values, and four Fourier series per track which model periodic fluctuations. The resultant synthetic wind values fluctuate around the monthly mean with both coupled and individual stochastic contributions. For more details on the track model method, readers should refer to Emanuel *et al*.^6^. This flow-driven movement is then modified by the addition of a beta-drift correction term **V**_*β*_, used to generate the movement of the cyclone each hour as:

1 $${{\rm{V}}}_{{\rm{track}}}=\alpha {{\rm{V}}}_{850{\rm{mPa}}}+\left(1-\alpha \right){{\rm{V}}}_{200{\rm{mPa}}}+{{\rm{V}}}_{\beta }$$

where *α* is a constant weight set to 0.8.

Rather than using a constant beta-drift correction as in Emanuel *et al*., the beta-drift here varies spatially, and is determined by a linear regression fitted on the large scale wind and latitude as suggested in Wu & Wang^17^ and Zhao *et al*.^18^ and implemented in Kelly *et al*.^19^. The tracks are simulated until one of the following conditions is met: the storm has lasted for 30 days; the track travels outside of the study domain (4°N – 50°N, 100°W – 5°W); or the intensity falls below a predefined threshold (see next section).

### Intensity

Every 6 hours, the TC intensity (10 m maximum sustained 1-minute wind speed) at the current track location is estimated using the deep learning model as developed in Xu *et al*.^12^. This model, trained on global Statistical Hurricane Intensity Prediction Scheme (SHIPS) predictors^20^ from 1982 to 2021, forecasts 6-hourly intensity changes in the North Atlantic using a multilayer perceptron with multiple feed-forward, fully connected layers. Each hidden layer computes a linear combination of the outputs from the preceding layer and applies a nonlinear activation function, enabling the model to effectively capture complex, nonlinear relationships among a multitude of predictors. The initial maximum wind speed is set to be 33 knots (17 m/s), the beginning of Tropical Storm designation according to the Saffir-Simpson scale. The original intensity model used the following 9 environmental variables to estimate the intensity change: the current intensity, the last 6-hour intensity change, vertical wind shear, 200 hPa zonal wind, maximum potential intensity, latitude, longitude, 1000 hPa equivalent potential temperature, and distance to major landmass. Since then, we have made further improvements to the model. First, we added two additional variables inspired by DeMaria *et al*.^20^ to account for the dry Saharan air layer and the westerly wind: low-level (850–700 hPa) relative humidity and the zonal component of the storm motion. Next, following Lee *et al*.^7^, the distance to major landmass predictor is replaced with the percentage of landmass within a 500 km distance from the storm center. The use of landmass percentage instead of distance to land helped correct some issues that were noted in the earlier version (e.g. storm intensity dropped too quickly when passing through the islands of Cuba and Hispaniola). While steering flow, vertical shear, storm longitude and latitude come from the track model, other variables like low-level relative humidity, maximum potential intensity and equivalent potential temperature come from the environmental conditions in ERA5 reanalysis.

Further, we also adopted the stochastic error component from the autoregressive intensity model described in Lee *et al*.^21^ and applied it to our deep learning model. The stochastic component works by randomly drawing errors from the training data, conditioned on the initial intensity. Since IBTrACS intensities are rounded to the nearest 5 kt interval and RAFT-predicted intensities are continuous, we obtain randomly drawn errors corresponding to the nearest two training intensity values. The probability of picking from either set of errors is based on the distance between the storm’s current intensity and the nearest two training intensities. For example, if we want to predict the intensity change for a model storm that is currently at 54 kt, the relevant training intensities would be 50 kt and 55 kt. In this situation, the model will draw an error randomly from the 55 kt bin $\frac{4}{5}$ of the time, and the 50 kt bin $\frac{1}{5}$ of the time. The final predicted 6-hour intensity change is then computed as the ML model’s predicted value plus this randomly drawn error correction.

While the track model uses monthly data to construct Fourier series as a way to generate random fluctuations, for intensity we use daily ERA5 reanalysis data^15^ to directly leverage daily variations of environmental conditions. To expose each synthetic TC to a variety of environmental conditions, a year between 1979 and 2018 is randomly assigned, and the ERA5 daily environmental conditions from that year are used as inputs. The intensity model continues along the track until the intensity drops below 25 kt. TCs lasting less than one day are considered transient and therefore removed from the dataset.

TC intensity is closely related to pressure differentials – specifically the low pressure at the storm center – and the radius of maximum winds (*R*_*max*_). Generally, as the central pressure decreases, TC intensity increases and *R*_*max*_ tends to contract, or decrease, thereby generating stronger winds and more severe weather conditions. To quantify the low pressure at the storm center, we fitted a linear regression between minimum sea level pressure and the maximum wind speed (*v*_*max*_) in knots. We trained the model using 2001–2016 North Atlantic data, and used 2017–2020 for testing. The model is able to explain 88% of the variance for the testing years, with a RMSE of 7.11 hPa. We compute pressure (hPa) given *v*_*max*_ as:

2 $$Pressure=1030.222-0.730\cdot {v}_{max}$$

Analytical wind generation also requires *R*_*max*_ as input, and here the *R*_*max*_ is calculated from storm intensity and latitude using a linear regression with logarithmic transformations fitted with 2001–2016 North Atlantic data, following Willoughby & Rahn^22^:

3 $${R}_{max}=48.7\cdot \exp \left(0.0163637\cdot {\rm{latitude}}-0.01450866\cdot {v}_{max}\right)$$

where *v*_*max*_ is in kt, and the resulting *R*_*max*_ is in nautical miles.

### Rainfall

TC-induced rainfall is simulated using the Tropical Cyclone Rainfall (TCR) model, a 4-component physics-based approach^13,14,23^. The theory behind the model is that the TC precipitation rate *P*_*rate*_ is proportional to the upward vapor flux, which can be obtained by multiplying the vertical velocity (*w*) above the boundary layer with the the 925–1000 hPa specific humidity *q*_*s*_. This *P*_*rate*_~*wq*_*s*_ relationship is theoretically and empirically validated in Lu *et al*.^14^, where a thorough derivation and justification of each step of the calculation can be found. The final precipitation (in mm/hr) is computed in the TCR model by multiplying this upward vapor flux *wq*_*s*_ by a constant precipitation efficiency *ε*_*p*_ and the ratio of the densities of air *ρ*_*air*_ to liquid water *ρ*_*liquid*_:

4 $${P}_{rate}={\varepsilon }_{p}\frac{{\rho }_{air}}{{\rho }_{liquid}}w{q}_{s}$$

A value of 0.0012 is used for the ratio of densities of air to water, and *ε*_*p*_ is set at 0.9. The vertical velocity (*w*) is estimated as the sum of contributions of four physical components (surface friction (*w*_*f*_), topography (*w*_*h*_), baroclinicity due to wind shear (*w*_*s*_), and vortex stretching (*w*_*t*_)):

5 $$w={w}_{f}+{w}_{h}+{w}_{s}+{w}_{t}$$

These components are estimated at each grid point over a 2-dimensional spatial domain as follows:

6 $${w}_{f}=-\frac{1}{r}\frac{\partial }{\partial r}\left({r}^{2}\frac{{\tau }_{\theta s}}{\partial M/\partial r}\right)$$

7 $${w}_{h}={\bf{V}}\cdot \Delta h$$

8 $${w}_{t}={H}_{b}\frac{1}{r}\frac{\partial }{\partial r}\left(r\frac{\partial M/\partial t}{\partial M/\partial r}\right)$$

9 $${w}_{s}=0.5fV\left({{\bf{S}}}_{200hPa-850hPa}\cdot {\bf{j}}\right)$$

The physical components are calculated using the following set of parameters:

- *r*, distance to the storm center
- *τ*_*θs*_, azimuthal surface stress (modeled as a function of surface drag coefficient and wind speed)
- *M*, angular momentum of wind
- V, wind velocity vector
- *h*, topographic height
- *H*_*b*_, representative depth scale of the lower troposphere
- f, Coriolis parameter
- ∂*M*/∂*t*, rate of change of the angular momentum with respect to time
- *S*_200*hPa*–850*hPa*_, wind shear computed as the difference between geostrophic wind and the 200 hPa and 850 hPa levels (V_*g*, 200hPa_–V_*g*, 850hPa_)
- j, unit vector pointing radially away from the center of the storm

The rainfall generation model relies on the synthetic surface wind field, which is generated analytically according to Holland *et al*.^24^ and as a function of *R*_*max*_. Analytical wind and rainfall are generated at hourly time steps, and hourly TC locations and intensities are linearly interpolated from the 6-hourly track and intensity simulations.

### Input data sources

Most atmospheric parameters are from ECMWF’s ERA5^15^ reanalysis, including large scale wind, relative humidity, wind shear, maximum potential intensity, equivalent potential temperature, and surface roughness (used in the frictional component of the rainfall model). US topographic elevation is derived from the USGS GMTED2010 project^25^.

## Data Records

The synthetic TC dataset^26^ generated by RAFT is publicly accessible at https://zenodo.org/doi/10.5281/zenodo.10392723, which includes 40,000 simulated storm events with each 6-hourly track location, along-track intensity (maximum wind speed and minimum pressure), and radius of maximum winds. The NetCDF4 file named “*RAFT.NA.v20231016.nc*” contains the complete set of variables for the synthetic TCs, as described in Table 1.

**Table 1:** Variables used in the RAFT dataset NetCDF file.

Variable	Unit	Explanation
basin_ID	—	Basin identification, 1 for North Atlantic
storm_ID	—	TC identification, starting from 0
year	—	The year of environmental conditions used for the intensity model and rainfall model
jday	—	Day of the year, ranging between 0 to 365
lon	°	Longitude
lat	°	Latitude
vmax	kt	Maximum wind speed
mslp	hPa	Minimum pressure
rmax	nmi	Radius of maximum wind

Additionally, for each synthetic event, we provide detailed data on storm-lifetime accumulated precipitation at each 0.08 by 0.08 degree grid point. This data is available in individual NetCDF4 files, named according to the convention “*modeled_rainfall_ERA5_syn_i.h5*”, where the *i* is the synthetic storm’s ID number. “*ERA5*” denotes the source of the reanalysis inputs, while “*syn*” clarifies that this file is for a synthetic track. Each of these files comprises five variables: total accumulated rainfall (*p_accum*) and its four constitutive vertical wind-induced rainfall components, frictional (*p_accum_f*), topographic (*p_accum_h*), shear (*p_accum_s*), and vortex stretching (*p_accum_t*), all measured in the unit of total millimeters of precipitation. During the calculation, each component of vertical wind is allowed to negatively contribute to the total precipitation, however in the final output of total precipitation and decomposition of accumulated precipitation, we convert any negative precipitation values to zeros to avoid misinterpretation. The rainfall data is presented on a regular spatial grid defined in “*RAFT_rainfall_latlon_grid.h5*”, which describes the grid in two variables, *lat* and *lon*.

## Technical Validation

### Spatial distribution of TCs

**Fig. 2:**
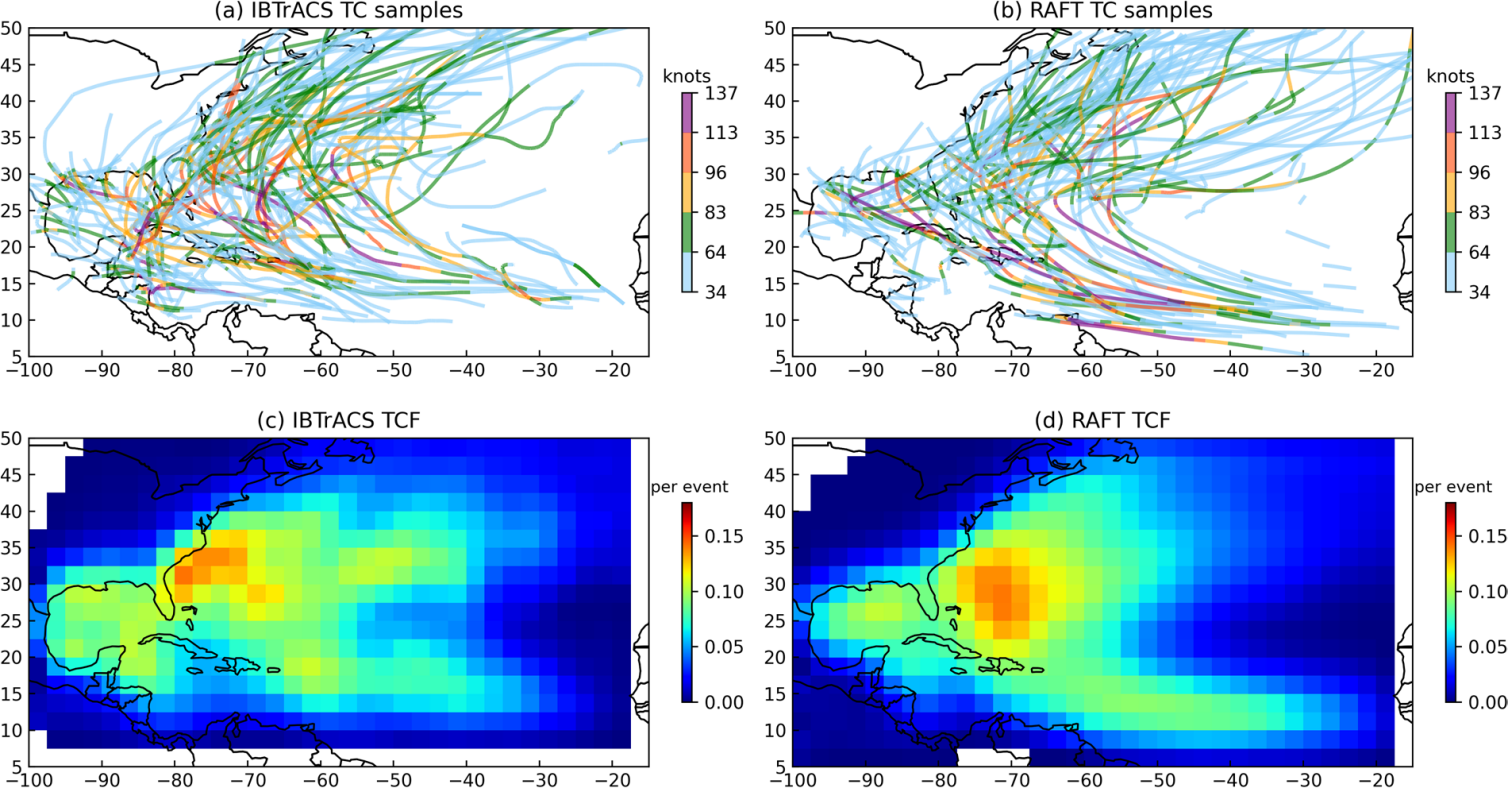
Comparative analysis of TC tracks and frequency in the North Atlantic. The upper panels depict 200 randomly sampled TC tracks from (**a**) IBTrACS and (**b**) RAFT. The lower panels depict the spatial distribution of TCF, calculated as the count of 6-hourly TC track locations per square 2.5 degrees normalized by the number of TC events for (**c**) IBTrACS and d) RAFT.

To assess the realism with which synthetic TCs are generated using RAFT, we begin by examining their spatial distribution. Figure 2a,b show 200 randomly selected TCs from observations and RAFT, respectively. Overall, the RAFT-simulated tracks match the observations from IBTrACS remarkably well, adequately capturing TC translation behavior in the Atlantic basin. There is a noticeable difference in the spatial distribution of sampled cyclogenesis points, where RAFT includes tracks initiated south of 10°N unlike what appears in the observations. This could partly be attributed to the methodology of RAFT cyclogenesis, which uses a 3-dimensional Gaussian kernel to expand the regions from where cyclones can originate beyond the observed subset of genesis points. RAFT tracks broadly tend to be clustered further offshore in the North Atlantic compared to observations, which is illustrated in the climatological track density or Tropical Cyclone Frequency (TCF) maps shown in Fig. 2c,d. TCF is defined as the number of 6-hourly TC track locations where the storm intensity exceeds 25 kt per square 2.5 degrees per TC event. Specifically along the coast, RAFT underestimates TCF when compared with observations, and landfalling TCs in RAFT are seen to dissipate sooner than observed TCs. This underestimation of track density near the coast is likely due to a systematic bias in the intensity model near land, which we plan to address in the future by developing a dedicated near-coastal intensity model. Despite these points, the spatial distribution of TCs is reasonably reproduced in RAFT, with a pixel-wise correlation coefficient of 0.87 (*p* < 0.001) when compared with observations.

### Salient features of TCs

**Fig. 3:**
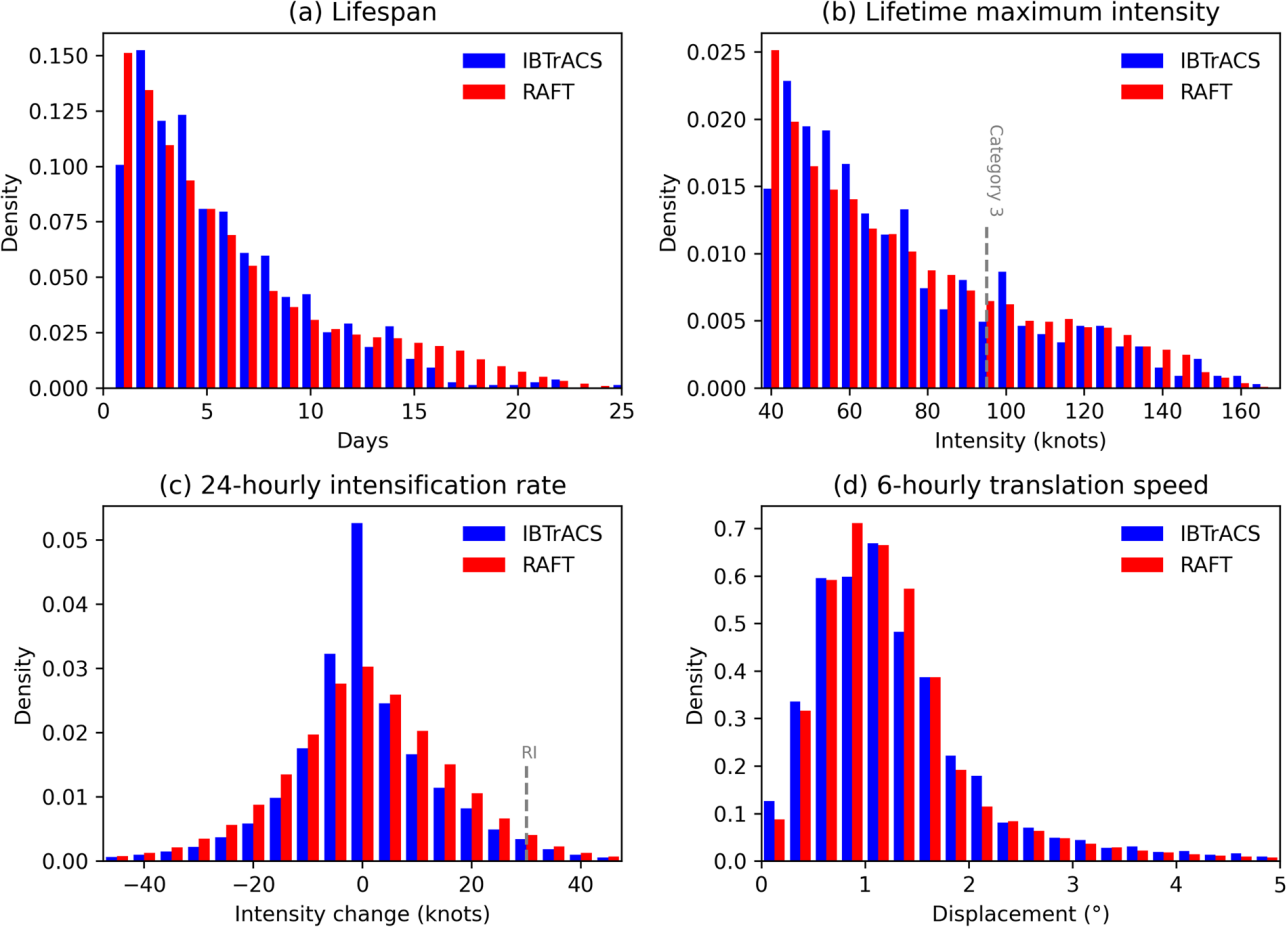
Histograms of IBTrACS (blue) and RAFT (red) for: (**a**) TC lifespan, (**b**) lifetime maximum intensity, (**c**) 24-hr intensity change, and (**d**) 6-hr translation speed. In each panel, the y-axis indicates the probability density, which is calculated so that the total area covered by the histogram adds up to 1. For observations, post-1970 data from IBTrACS is used. All 40,000 synthetic TCs from RAFT are used.

To further understand the simulation of TCs using RAFT, we now explore the representation of TC properties particularly relevant for impacts. RAFT captures the duration of TCs remarkably well, as illustrated in the lifespan distribution in Fig. 3a. Distributions of TC translation speeds (Fig. 3d) indicate that RAFT TCs move at comparable speeds to observed TCs, as indicated by the mean 6-hourly displacements for RAFT and observations which are 1.33° and 1.27°, respectively (Table 2). The third important aspect of TCs that we consider here is the intensification rate (Fig. 3c), defined as the change in TC intensity over a 24-hr period. The TC intensification rate distributions are remarkably consistent between the RAFT simulations and observations. Importantly, RAFT well represents the occurrence of TC rapid intensification (RI), defined as an instance where a storm increases in intensity by 30 kt or higher in 24 hours. RI occurs roughly 3.4% of the time in RAFT, closely aligning with the 3.8% occurrence rate in observational data. This is a major advantage of RAFT, considering the difficulty associated with simulating RI even for high-resolution dynamical models^27^.

**Table 2:** Mean and standard deviation values for TC characteristics in the IBTrACS and RAFT datasets over the North Atlantic basin.

	IBTrACS mean	IBTrACS std	RAFT mean	RAFT std	# of std from IBTrACS mean
Translation, 6-hourly displacement (°)	1.27	0.81	1.33	0.95	0.06
Life span (day)	5.70	4.18	6.12	5.12	0.10
Lifetime max wind (kt)	68.09	29.12	64.88	31.52	−0.11
wind speed (kt)	53.24	24.37	54.88	24.93	0.07
Lifetime min pressure (hPa)	979.44	24.54	982.84	23.03	0.14
Pressure (hPa)	992.02	18.94	990.14	18.21	−0.10
Radius of max wind (nmi)	45.28	31.12	36.86	12.85	−0.27

The time period 1970–2021 is used for the IBTrACS dataset.

Next, we compare distributions of TC lifetime maximum intensity (LMI), defined as the peak one-minute sustained wind speed reached during the lifetime of a TC. The PDF of LMI for RAFT simulations similarly resembles the LMI of observed TCs (Fig. 3b), including for storms that reach Category 3 and higher strength. This strong agreement between RAFT simulations and observations is further illustrated in Table 2. The mean LMI for RAFT simulations is 64.88 kt, similar to the observed mean LMI of 68.09 kt. Similarly, the mean maximum wind speed simulated in RAFT is 54.88 kt, closely matching the observed mean of 53.24 kt. However, there is a noticeable disparity in the mean TC radius of maximum winds (*R*_*max*_), with RAFT’s average of 36.86 nautical miles reflecting a 19% underestimation when compared with the corresponding observed mean *R*_*max*_ of 45.28 nautical miles, as detailed in Table 2. Lastly, the mean lifetime minimum central pressure is 982.84 hPa, remarkably similar to the observed value of 979.44 hPa.

### Coastal TC characteristics

**Fig. 4:**
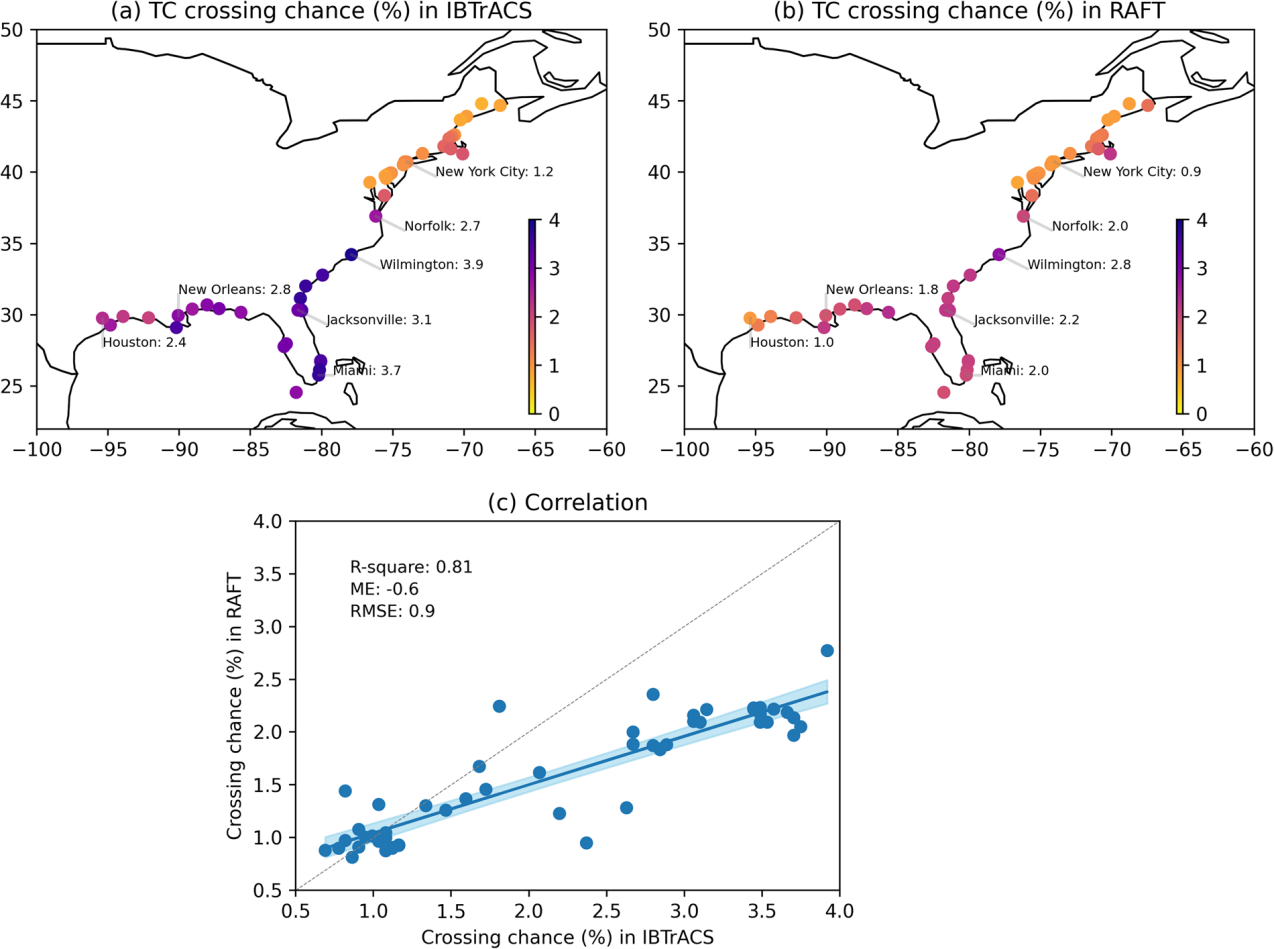
TC crossing probability for 51 U.S. coastal cities for (**a**) observations and (**b**) RAFT. Each TC crossing is counted if the track passes within a 1 degree radius of the city center coordinate, and the TC crossing probability is calculated as the fraction of TCs making the crossing, including both direct landfall from the ocean and recurving from land. For observations, post-1970 data from IBTrACS are used. For RAFT, all 40,000 synthetic TCs are used. (**c**) Correlation plot of TC crossing chance between observations (x-axis) and RAFT simulations (y-axis) for 51 major U.S. coastal cities. The blue line depicts the best-fit regression line, and the shading denotes the 95% confidence interval of the linear regression using the bootstrap method resampled 10,000 times. Displayed metrics include the corresponding R-squared value, ME and RMSE. The dashed grey line represents the 1:1 perfect correlation line.

**Fig. 5:**
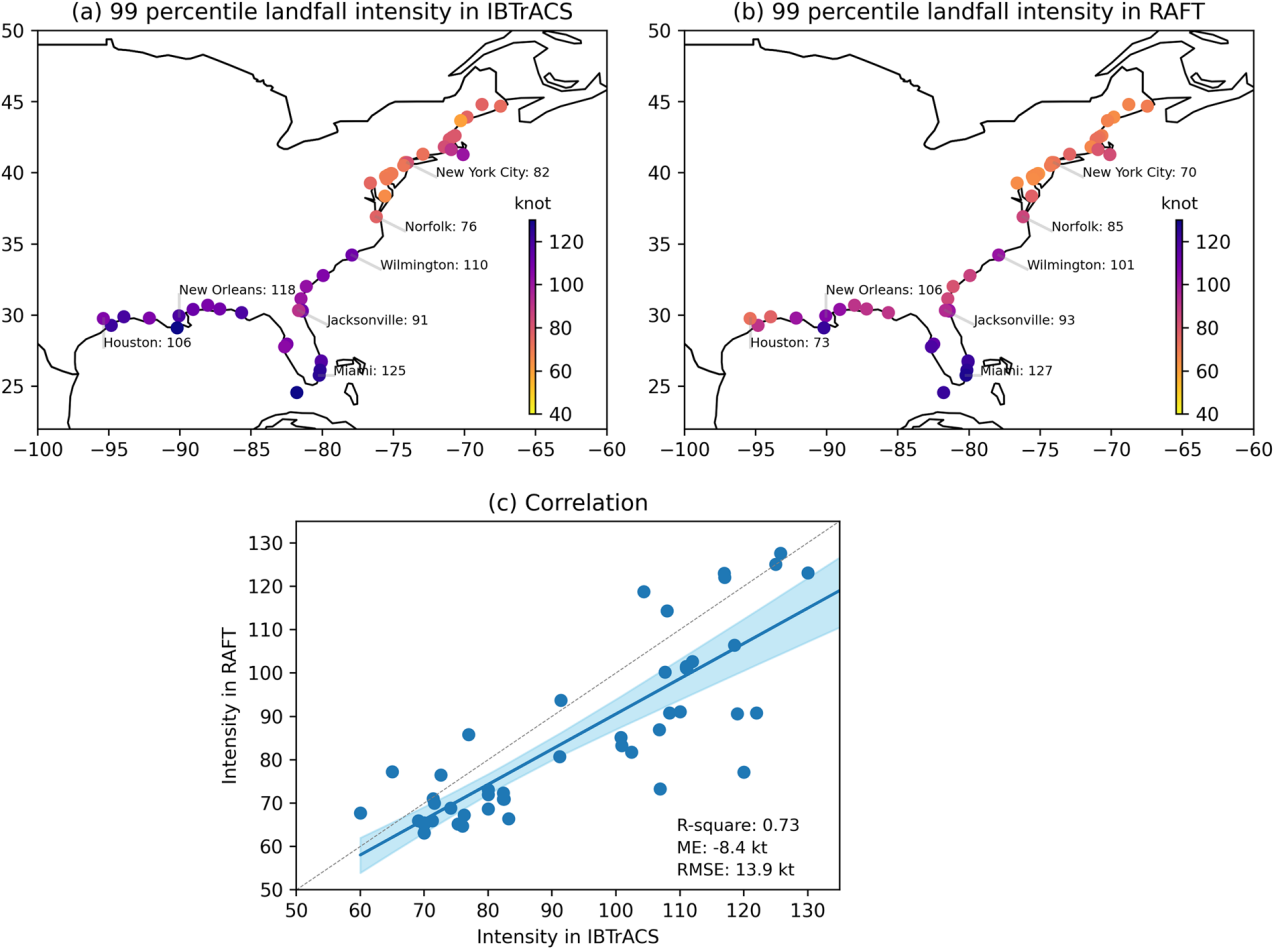
Extreme TC winds for 51 major U.S. coastal cities for (**a**) observations and (**b**) RAFT. Extreme TC winds are defined as the 99^*th*^ percentile value of the TC wind distribution at a location. For observations, post-1970 data from IBTrACS are used. For RAFT, all 40,000 synthetic TCs are used. (**c**) Correlation plot of extreme TC winds between the observations (x-axis) and RAFT-simulations (y-axis) for 51 U.S. coastal cities. The blue line depicts the best-fit regression line, and the shading denotes the 95% confidence interval of the linear regression based on the bootstrap method resampled 10,000 times. Displayed metrics include the corresponding R-squared value, ME and RMSE. The dashed grey line represents the 1:1 perfect correlation line.

So far, we have examined some basin-scale aspects of TCs simulated in RAFT. However, one main goal of producing the RAFT-based synthetic TC dataset is its potential use for risk assessment. Therefore, we will now focus our discussion on TC characteristics in coastal regions. Figure 4a depicts the observed TC crossing probability for 51 major U.S. cities. Historically, more TC crossings have occurred over the US Southeast Coast, followed by the Gulf Coast and the Northeast Coast. The spatial distribution of TC crossing is reasonably reproduced in RAFT (Fig. 4b) and is in broad agreement with observations despite some differences. For instance, RAFT tends to underestimate landfall over the Southeast Coast and the Gulf Coast. RAFT is however able to capture 81% of the observed variance in TC crossing probability for major U.S. cities, thereby modeling the spatial distribution of TC landfall and recurving fairly well (Fig. 4c). Next, we investigate the ability of RAFT to accurately represent TC winds in US coastal regions. Since extreme TC winds have the strongest impacts, here we consider the 99^*th*^ percentile TC winds upon landfall. The observed distribution of extreme TC winds reveals that most regions along the northern Gulf Coast, Florida peninsula and the Southeast US Coast have historically experienced the most devastating TC winds exceeding 90 kt, which is close to Category 3 strength (Fig. 5a). When considering the corresponding distribution produced by RAFT, we see that RAFT underestimates the magnitude of extreme TC winds by about 8 kt (Fig. 5b), particularly over the Gulf and Southeast Coasts. Despite this general underestimation, RAFT adeptly simulates several instances of intense TC landfalls. For example, extreme TC winds impacting New Orleans are close to Category 3 strength, and exceed Category 4 strength for Miami in both the observations and RAFT simulations. Overall, the spatial distribution of extreme TC winds (Fig. 5c) is well represented in RAFT with an R-squared value of 0.73 when compared with observations.

Finally, we evaluate TC-induced rainfall in coastal regions based on RAFT using daily rainfall data from the Global Historical Climate Network Daily Database (GHCN)^28^ rain gauges. We select GHCN rain gauges within 2° of the US Atlantic and Gulf Coasts that have complete recordings of daily rainfall since 1979. Since rain gauge measurements are prone to underestimation due to the high winds associated with TC events, rain gauge measurements are bias-corrected using the wind-correction function by the World Meteorological Organization^29^. Observed TC tracks from 1979 to 2018 are then used to estimate which precipitation is TC-induced, where a daily rainfall recording is considered to be TC-induced if an observed TC track passes within 500 km of the gauge during that day. We compare these TC rainfall observations with those simulated by RAFT. Despite potential limitations (e.g., observed precipitation within 500 km of a TC may have been generated by other storm systems, and larger TCs can induce precipitation outside of a 500 km radius), this method provides an indication of where RAFT may systematically over- or underestimate TC-induced rainfall.

**Fig. 6:**
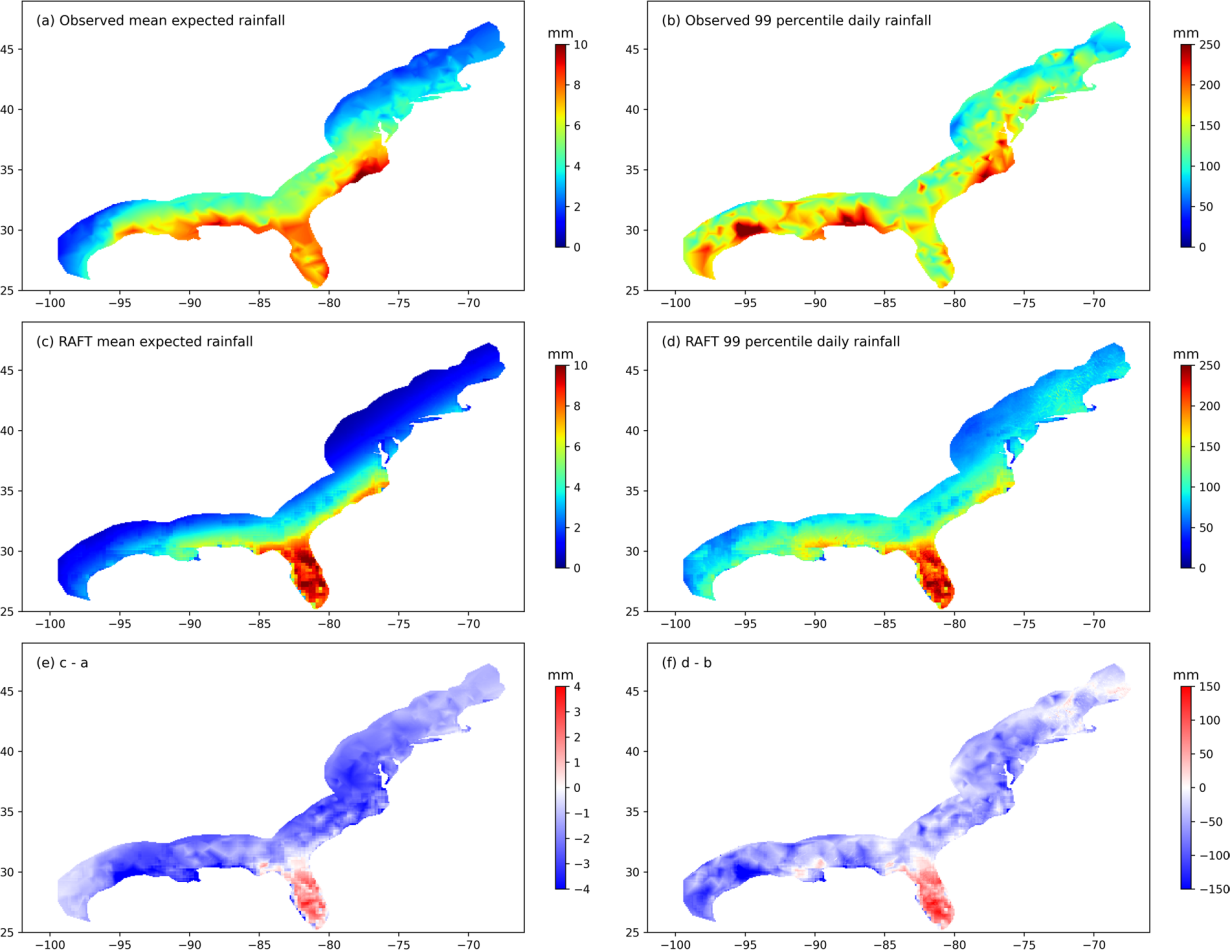
(**a**) Observed and c) RAFT-simulated mean expected TC rainfall per event (mm). The difference plot between (**a,c**) is illustrated in (**e**). Panels (**b**), (**d**), and (**f**) are the same as (**a**), (**c**), and (**e**) but depicting extreme TC rainfall (mm), defined as the 99^*th*^ percentile value of TC-induced daily precipitation at a location. While the observations are based on daily rainfall data from GHCN rain gauges, the modeled data is based on RAFT-simulated TC rainfall.

**Fig. 7:**
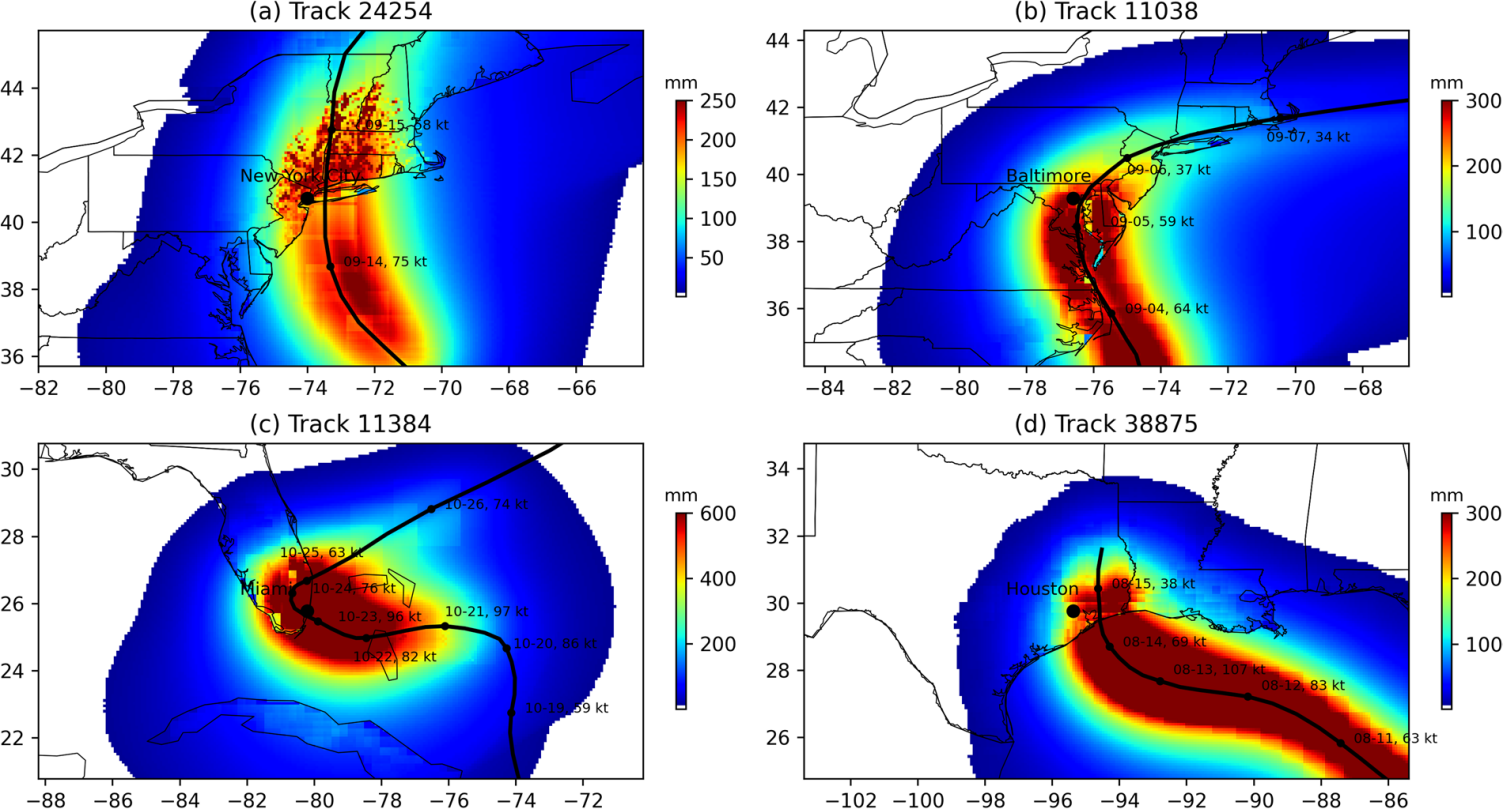
Sample tracks with along-track accumulated precipitation (mm) and intensity (kt). TC tracks are denoted by a solid black line, and discrete intensity values with date information are provided along the track. Subpanels represent TC tracks passing near: (**a**) NYC, NY (**b**) Baltimore, MD (**c**) Miami, FL (**d**) Houston, TX.

Figure 6a shows the observed climatology of mean expected rainfall per event, a metric that represents the combined influence of TC track spatial distribution, storm intensity, and rainfall efficiency. TCs tend to produce more rainfall over the northern Gulf Coast, Florida peninsula and the Southeast Coast, and less rainfall over the Northeast Coast and the western Texas Coast. RAFT is able to broadly capture this spatial pattern (Fig. 6c) albeit with a weaker magnitude. In other words, RAFT broadly underestimates TC rainfall everywhere except for over the Florida peninsula (Fig. 6e). The mean error is −1.7 mm, with an undercatch of about 35%. This underestimation is likely due to the underestimation of coastal TCF and landfall intensity, as well as bias from the rainfall model. Despite this, RAFT-predicted mean expected rainfall has a statistically significant pixel-wise correlation coefficient of 0.86 (*p* < 0.001) when compared with observations, indicating that RAFT effectively captures mean expected rainfall spatial patterns within 2° from the coast. We also evaluate extreme rainfall from TCs using 99^*th*^ percentile daily rainfall. The spatial pattern of extreme TC rainfall (Fig. 6d) simulated by RAFT is broadly similar to the RAFT-simulated mean expected rainfall (Fig. 6c), with a high correlation coefficient of 0.95. However, the observed pattern of extreme TC rainfall (Fig. 6b) is patchy with larger values more widely distributed, having a correlation of only 0.60 with observed mean expected rainfall (Fig. 6a), indicating that extreme rainfall patterns may be more complex than what TCR is capable of modeling. Observed extreme TC rainfall appears more patchy than what is simulated by RAFT likely due to localized extreme events in the IBTrACS dataset. For example, the observed maxima occurs near the Texas Coast, and is likely associated with the torrential downpour from TC Harvey (2017). The difference map of extreme TC rainfall (Fig. 6f) further indicates that RAFT underestimates the magnitude over almost the entire coast except for the Florida peninsula. The mean bias in expected 99^*th*^ percentile daily rainfall simulated by RAFT is −39 mm (underestimation by 27% of the observed mean) and the pixel-wise correlation between RAFT-simulated 99^*th*^ percentile daily rainfall and that of observations is relatively lower (*r* = 0.39, *p* < 0.001). This broad underestimation and spatial bias is likely attributed to TC rainfall simulation challenges in addition to RAFT’s underestimation of TC frequency and winds upon landfall, noted previously (Figs. 4b, 5b). Nevertheless, RAFT is able to simulate some extreme rainfall events near major coastal cities, as illustrated in Fig. 7 with their respective along-track intensity and accumulated precipitation.

## Usage Notes

RAFT is a computationally efficient framework that can be used to simulate numerous synthetic TCs along with their properties. Here, we utilize RAFT to generate 40,000 synthetic TCs reflecting the current North Atlantic TC climatology using climate conditions of the last 40 years^26^. TC tracks, along-track intensity (maximum wind speed and minimum sea level pressure), radius of maximum winds and TC-induced rainfall are provided for comprehensive TC hazard assessment. The RAFT-generated dataset presented here enables the study of several downstream risks pertinent to the TC risk modeling community, including storm surge, freshwater flooding, wind hazard, and electrical power outages. The rainfall component of RAFT, which is not included in many similar approaches^6–8^, makes our dataset unique and valuable for assessing TC flood risk.

In the pursuit of generating synthetic TCs, it is imperative to critically examine the inherent limitations, assumptions, and potential drawbacks associated with various model components that collectively constitute the RAFT model. The genesis and track models used here follow Emanuel *et al*.^6^ by generating storms as a random draw from the probability distribution of historical cyclone genesis points, then moving storms according to a weighted average of the ambient flow plus beta-drift. Notably, the track method employed here cannot capture the effects of nonlinear interactions between tropical and extratropical systems, therefore caution should be exercised in these cases. Since the beta-drift term is obtained from a specific pattern of large-scale environmental flows, adaptations may be necessary when applying this relationship to future climate scenarios. The intensity model used here has been trained on global data and thus may be extended globally, while at this time it has only been tested in the Atlantic basin. Additionally, as with any deep learning model, performance is expected to be better in regions with more frequent TC events than those with sparse events, which in part might explain the intensity model’s land-ocean bias. Lastly, the rainfall model implemented in RAFT is the physics-based TCR model^13,14,30^ which we find to broadly underestimate TC-induced rainfall along the Atlantic and Gulf Coasts, and overestimate rainfall over the Florida peninsula. It’s important to highlight that this model assumes a fixed precipitation efficiency and is highly sensitive to the drag coefficient *C*_*d*_, performing less satisfactorily when representing regions with large gradients in surface roughness^23^. Xi *et al*.^30^ found that TCR overestimates rainfall at the core and underestimates rainfall outside of the core, and other studies have found that TCR may not handle extratropical transition very well^6,14,23^, thus RAFT track and precipitation accuracy is expected to be best in areas where TCs have not yet transitioned to extratropical storms. Overall, the ability of these models to produce a multitude of synthetic TCs makes RAFT a valuable tool for advancing our understanding of TCs and improving preparedness for severe weather events.

RAFT excels in simulating spatial variability in TC characteristics relevant for risk, such as TCF and the distribution of landfall locations. It also effectively captures basin-scale TC characteristics like translation speed, intensity, and rapid intensification. However, RAFT tends to underestimate extreme winds at landfall (Fig. 5b) and TC-induced precipitation over coastal regions (Fig. 6). Given these limitations, caution is advised when interpreting extreme wind and rainfall results at local-to-regional scales. Statistical bias-correcting techniques, such as quantile mapping^31^, can be employed to overcome these limitations and improve the potential of this data for risk assessment. While the track and intensity components of RAFT are interconnected, the framework offers flexibility for users, allowing the implementation of an alternative rainfall model suiting their requirements. To further address the challenges of simulating extreme rainfall near the coast, we are currently developing a deep-learning rainfall model specifically tailored for coastal risk assessment. Additionally, ongoing research efforts are focused on improving the spatial representation of storms and evaluating their impacts on risks such as urban flooding, power outages and damage to infrastructure. Our approach is also adaptable for future climate scenarios since RAFT can be driven by climate model simulations, and our upcoming work includes releasing RAFT simulations driven by CMIP6 future climate scenarios. Moreover, as RAFT is currently confined to the North Atlantic, plans are underway to expand its domain and increase the capability of RAFT to simulate synthetic TCs on a global scale.

## Data Availability

Code to read and process the RAFT synthetic TC dataset can be found at 10.5281/zenodo.7976242.
