# Publisher Correction: A North Atlantic synthetic tropical cyclone track, intensity, and rainfall dataset

**DOI:** 10.1038/s41597-024-03040-6

**Published:** 2024-02-06

**Authors:** Wenwei Xu, Karthik Balaguru, David R. Judi, Julian Rice, L. Ruby Leung, Serena Lipari

**Affiliations:** Pacific Northwest National Laboratory, Richland, 99354 USA

**Keywords:** Natural hazards, Atmospheric dynamics, Energy security, Natural hazards, Atmospheric dynamics, Energy security

Correction to: *Scientific Data* 10.1038/s41597-024-02952-7, published online 25 January 2024

The copyright holder for this article was incorrectly given as ‘The Author(s)’ but should have been ‘Battelle Memorial Institute’. The original article has been corrected.

